# Partial Pulpotomy in Mature Permanent Molars with Symptoms Indicated Irreversible Pulpitis Using MTA: A Study of Three Case Reports over Four-Year Follow-Up

**DOI:** 10.1155/2023/1344101

**Published:** 2023-10-17

**Authors:** Rami Zenaldeen, Rami Kaddoura, Hasan Alzoubi, Hassan Achour, Ossama Aljabban

**Affiliations:** ^1^Department of Endodontics and Operative Dentistry, Faculty of Dentistry, Damascus University, Damascus, Syria; ^2^Department of Pediatric Dentistry, Faculty of Dentistry, Damascus University, Damascus, Syria

## Abstract

VPT is vital pulp therapy, a biologically based procedure that combines several therapeutic techniques to maintain the entire or a portion of the dental pulp. Interest in VPT has grown due to recent developments in bioactive materials and an understanding of biological pulp reparative responses. This case report is aimed at evaluating the success rate of partial pulpotomy in permanent molars with symptoms indicating irreversible pulpitis using MTA and presenting with extremely deep carious lesions over four years of follow-up. All patients came with spontaneous and severe pain. Each tooth was isolated with a rubber dam and disinfected with 5.25% NaOCl before caries excavation. After caries removal, a partial pulpotomy was performed on 2–3 mm of the exposed pulp. Bleeding time was recorded after hemostasis was achieved, and then MTA was placed over the exposed pulp. The permanent restoration was placed after pulp capping, and postoperative periapical radiographs were taken. Patients were scheduled for clinical and radiological examinations for four years based on 6-month intervals. All teeth revealed a successful outcome throughout the follow-up periods (clinically and radiographically) with complete resolution of clinical signs and symptoms. Partial pulpotomy using MTA might be an effective long-term management strategy for permanent molars clinically diagnosed with irreversible pulpitis.

## 1. Introduction

Spontaneous and intense pain is typically a sign of irreversible pulpitis. However, several histological studies showed that in these cases, a good portion of coronal pulp tissue was normal and uninflamed. At the same time, the inflammatory process in these teeth is restricted to the coronal part directly underneath the carious lesions unless they are long-standing [1, 2]. Hence, by removing only the inflamed and infected pulp tissue under carious exposure, it may be possible to preserve irreversibly inflamed pulps [3]. This has altered treatment options to manage irreversible pulpitis [4].

Root canal therapy (RCT) is the conventional treatment for permanent teeth with irreversible pulpitis [5]. It is, however, a moderately time-consuming choice that can be both expensive and nonbiological [6]. Moreover, it is found that although RCTs for vital pulp teeth yield considerable success rates, some studies have shown that high prevalence of insufficient root canal fillings and apical periodontitis, indicating the RCT expected outcomes are not regularly attained. [7, 8].

VPT aimed to preserve the vitality of the dental pulp after removing as little pulp tissue as possible and sealing the pulp wound with biocompatible material [9, 10]. VPT consists of direct or indirect pulp capping and pulpotomy (partial or full) [11]. Its indications have long been restricted to the treatment of pulpitis in deciduous teeth and permanent immature teeth [12–14]. Over the last two decades, the advances in our understanding of the reparative capacity of dental pulp [15, 16], as well as the introduction of bioactive calcium silicate-based cement [17, 18], have led to the recent emergence of VPT as a promising treatment option even in teeth with symptomatic irreversible pulpitis. [9]. This paper aimed to investigate the effectiveness of partial pulpotomy in three cases, presenting symptoms indicative of irreversible pulpitis and deep carious lesions.

## 2. Case Presentation

### 2.1. Case 1

**Figure 1:**
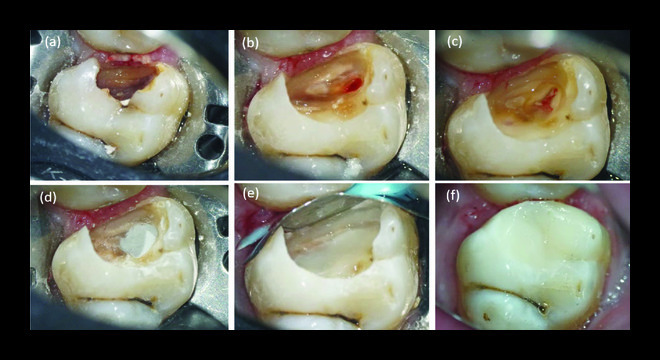
Clinical photographs. (a) Before caries removal, (b) carious pulp exposure, (c) pulpotomy level and hemostasis, (d) MTA was placement, (e) base of RMGIC, and (f) Final restoration.

**Figure 2:**
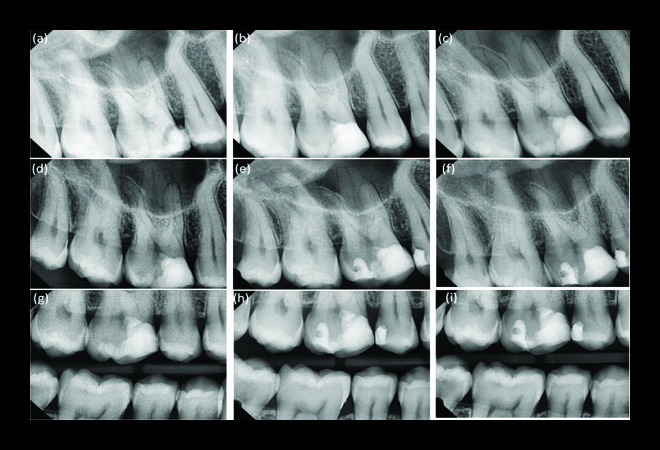
(a) Diagnostic periapical radiograph. (b) After partial pulpotomy. (c–f) 6-, 12-, and 18-month postoperative periapical radiographs, respectively, showing normal periapical tissues. (g) Two-year postoperative bitewing radiograph showing caries in the distal occlusal surface. (h, i) Three-year and four-year postoperative bitewing radiographs, respectively, showing no evidence of mineralized bridge formation.

A male patient (28 years old) presented to the Department of Endodontics and Restorative Dentistry, Damascus University, complained of spontaneous pain in the upper right quadrant and severe pain that persisted after removing the thermal stimulus. The response was reproduced on cold testing (Endo Ice; Coltene, Altstätten, Switzerland). Clinical examination showed a large carious lesion (in the occlusal and mesial surfaces) affecting the maxillary right permanent first molar (tooth 16) was identified (Figure 1(a)). No related sinus tract was identified surrounding the affected tooth, and periodontal probing revealed no abnormalities. There was also no percussion discomfort. Radiographic examination showed an extremely deep carious lesion (Figure 2(a)). Clinical, radiographic, and pulp sensibility examination results led to the diagnosis of symptomatic irreversible pulpitis in this case. The diagnostic challenge was a clinical diagnosis of the vital inflamed pulp that can heal based on subjective and objective criteria.

The risks of both treatments (RCT and VPT) were explained to the patient. He chose VPT and signed the consent and informed consent forms.

After local anesthetic and placement of a rubber dam, the tooth was cleaned with 5.25% sodium hypochlorite. Dental caries were removed using a sterile round bur on a slow-speed handpiece, and after the pulp exposure (Figure 1(b)), 2 to 3 mm of the pulp was removed using a high-speed handpiece underwater coolant (Figure 1(c)). The cries cavity was rinsed using 2.5% NaOCl solution, and the pulp tissues were evaluated under the pulpotomy level at 3x magnification (Dental Loupe, Eighteeth, China). The pulp tissues were considered normal if they were reddish-pink colour and had light red bleeding. A cotton pellet soaked in 2.5% NaOCl was applied to achieve hemostasis (hemostasis was achieved in 4 minutes) (Figure 1(c)). Then, 2-3 mm of MTA (PD-MTA, Switzerland) was applied over the pulp wound (Figure 1(d)) using the MapOne system (Verey, Switzerland). After 15 minutes, RMGIC (resin-modified glass-ionomer cement (Fuji II LC; Japan)) was applied, and direct resin composite was applied (Tetric-Ceram, Ivoclar Vivadent) using the sectional precontoured metal matrix (Palodent system standard matrices, Dentsply).

A periapical radiograph was taken postoperatively, and the patient underwent four years of follow-up visits every six months (Figures 2(c)–2(i)). The patient was pleased that the treatment was carried out without root canal treatment and in the absence of clinical symptoms.

### 2.2. Case 2

**Figure 3:**
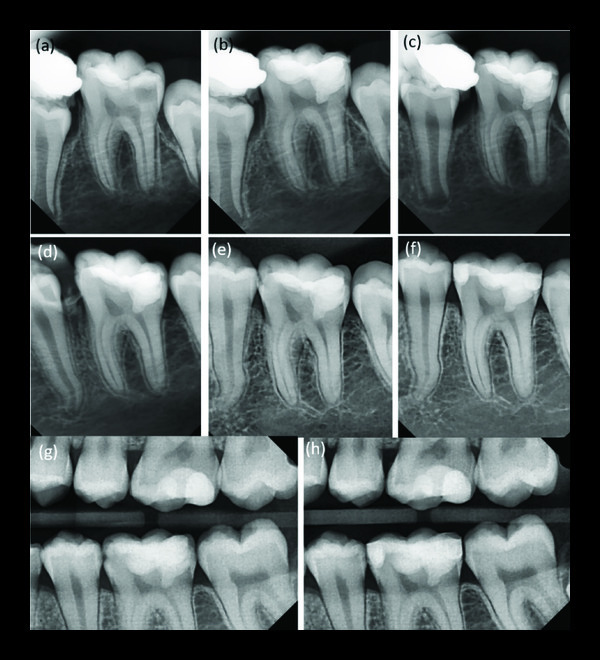
(a) Diagnostic periapical radiograph, (b) after partial pulpotomy, (c) after six months, (d) one-year follow-up showing mineralized bridge formed underneath capping material and normal periapical tissues, (e) 18-month follow-up, (f) two-year follow-up, (g) three-year postoperative bitewing radiograph showing mineralized bridge formed under capping material and new caries at distal and mesial surfaces, and (h) four-year postoperative bitewing radiograph.

A female patient (9 years old) presented to the Department of Endodontics and Restorative Dentistry, Damascus University, complaining of sharp and spontaneous pain in the mandibular left posterior region. Clinical examinations revealed that tooth no. 36 (mandibular left permanent first molar) showed a deep occlusal carious lesion. A sensitivity test using Endo Ice (Endo Ice; Coltene, Altstätten, Switzerland) showed pain lingering for several minutes after the cold stimulus was removed. Radiographic examination revealed a deep carious lesion extending to the distal horn of the pulp (Figure 3(a)). Clinical, radiographic, and pulp sensibility examination results led to the diagnosis of symptomatic irreversible pulpitis in this case. The diagnostic challenge was a clinical diagnosis of the vital inflamed pulp that can heal based on subjective and objective criteria.

The patient and her legal guardian were informed of the partial pulpotomy with MTA treatment option as an alternative to RCT. Her legal guardian provided the written approval. The patient received the same pulpotomy treatment with MTA as in case 1. The patient legal guardian called back for follow-up visits every six months for a total of four years (Figures 3(c)–3(h)). The patient legal guardian was pleased that the treatment was carried out without root canal treatment and in the absence of clinical symptoms.

### 2.3. Case 3

**Figure 4:**
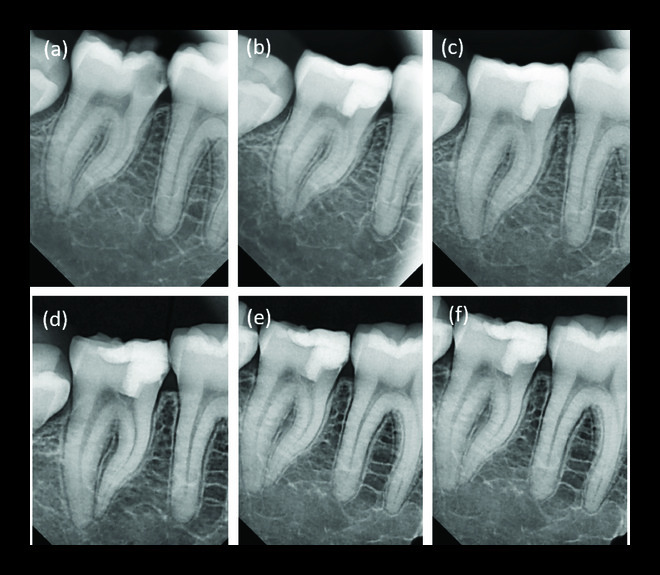
(a) Diagnostic periapical radiograph. (b) After partial pulpotomy. (c–f) One-year, two-year, three-year, and four-year postoperative periapical radiographs, respectively, showing normal periapical tissues and mineralized bridge formation.

A female patient (19 years old) presented to the Department of Endodontics and Restorative Dentistry, Damascus University because of severe spontaneous pain that awakened her at night and could only be reduced by analgesics. Clinical examination showed a carious lesion in the mandibular left permanent second molar (tooth 37) and a slight tenderness on percussion. Radiographic examination showed a deep carious lesion (Figure 4(a)). The case was diagnosed as irreversible pulpitis based on the clinical and radiographic findings. The diagnostic challenge was a clinical diagnosis of the vital inflamed pulp that can heal based on subjective and objective criteria. The mode of partial pulpotomy modality using MTA was clarified to the young patient, and her written consent followed this step. The same pulpotomy stages performed in our aforementioned cases were applied to this 19-year-old female patient. For the coming four years, she was instructed to admit six-month-interval follow-ups (Figures 4(c)–4(f)). The patient was pleased that the treatment was carried out without RCT and in the absence of clinical symptoms.

### 2.4. Follow-Up

Clinical failure evaluations include soft tissue swelling, pathological tooth mobility, spontaneous or stimulant pain, fistula, and tenderness to percussion. Radiographical success evaluations included the absence of periapical and furcation radiolucency, the absence of pathological internal or external root resorption, no widened periodontal ligament space, and dentin bridge formation.

The follow-up periods for the first and third clinical cases were 6, 12, 24, 36, and 48 months. The second case was evaluated at 6, 12, 36, and 48 months because the patient could not be contacted for a 24-month follow-up. At every follow-up appointment, the clinical follow-up evaluation of three cases showed that the teeth were still functional, had a positive response to the cold test, had no symptoms of pulpitis, had normal probing pocket depth, and had normal mobility.

In addition, the radiographic examination of the teeth showed no radiolucency (periapical and furcal) and no internal or external root resorption (Figures 2–4). Moreover, the radiographs showed mineralized bridge formation in cases 2 and 3 (Figures 3 and 4), while no bridge formation was observed in case 1 (Figure 2).

**Figure 5:**
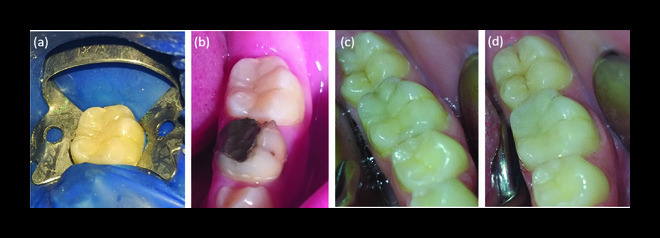
Intraoral photographs. (a, b) Composite resin restoration immediately after MTA partial pulpotomy for case 2. (c) The tooth after three-year postoperatively with new caries at the distal and mesial surface. (d) Tooth after removing new caries and restoring them with composite resin.

The tooth in case 1, after 24 months, required an additional restorative intervention with composite resin due to the progression of previously arrested caries in the distal occlusal surface (Figure 5). Similarly, the tooth in the case 2 underwent an additional restorative procedure due to having new caries at the distal and mesial surfaces after 36 months. Despite this, these teeth remained functional and did not show any signs or symptoms of any disease at any point.

## 3. Discussion

Partial pulpotomy is defined as the removal of 2-3 mm of the exposed pulp, followed by biocompatible material to seal the pulp wound [11]. As the superficial layer of infected tissue was removed, partial pulpotomy is the first treatment choice for carious pulp exposure [19, 20]. The clinical studies about the success of partial pulpotomy procedure in carious pulp exposure even when there are symptoms of irreversible pulpitis are low, and more long-term data are needed [19–22].

Managing carious exposure with symptomatic irreversible pulpitis requires the removal of coronal pulp tissue either partially or completely [20, 23]. In this case report, all teeth were diagnosed with irreversible pulpitis and had deep carious lesions that penetrated the dentine thickness. Hence, removing the carious tissue non-selectively rather than selectively in all cases included in the present case reports was decided.

A detailed assessment of the pulp tissue's inflammatory status is essential for the VPT to be successful. Unfortunately, the weak link between symptoms and the pulp's histological condition, this evaluation is more predictive [2, 24]. Because histological assessment of pulp tissues is not possible in clinical settings, several partial pulpotomy studies have relied on the pulp tissues' appearance and hemostasis time to determine the condition of the pulp and the treatment prognosis [20, 21]. The extent of inflammation within the dental pulp and determining the most appropriate treatment can be made through examination of the pulp tissues' appearance under carious exposure as well as the hemostasis time [3]. Hence, it has been advised to utilize magnification to improve the exposed pulp management [11]. After pulp amputation, a magnified inspection of the pulp wound using an operating microscope or even dental loupes under illumination may be beneficial and provide a more detailed view of the injured tissues. [3].

Although hemostasis achievement is essential for VPT success, clinical studies have revealed no significant association between hemostasis times (within 1-10 minutes) and the success of VPT [20, 22, 23]. Nevertheless, advanced pulpal inflammation may cause pulpal bleeding that is difficult to stop [25]. Therefore, the treatment strategy should be changed, moving from partial pulpotomy to full pulpotomy or RCT, if bleeding cannot be controlled despite efforts at hemostasis. [3]. For the teeth included in this case reports, the pulp wound underneath the level of amputation was thoroughly inspected under magnification and illumination. The pulp tissues showed a normal appearance, texture, and color. In cases 1, 2, and 3, the bleeding was controlled within four minutes, two minutes, and five minutes, respectively.

In every case in the current case reports, 2.5% NaOCl was used as a lavage solution to help achieve hemostasis while also disinfecting the pulp wound and cavity. NaOCl exhibits unique properties, including a high efficiency against bacteria and tissue-dissolving effects on necrotic soft tissue [26], which may minimize the need for mechanically removing infected tissue [27].

The ideal capping material for VPT should immediately seal off any bacterial leakage, promote healing of the remaining pulp tissues, and have bioactive characteristics that initiate the associated biological processes involving dentin bridge formation [28]. Calcium hydroxide (CH) was the gold standard for pulp capping, but it has shown varied and unpredictable results, with success rates decreasing over time [29]. Calcium silicate-based materials, such as MTA, have exhibited better clinical outcomes when used to treat exposed pulp compared to CH [30, 31]. These materials also produce thicker, higher-quality histological mineralized bridges than CH [18]. It has been demonstrated that tricalcium silicate materials can promote cellular differentiation, release dentin matrix components, and mineralization which increase reparative processes in dental pulp cell populations. [32]. In light of these characteristics, MTA was chosen to be used in this study.

Pulp tissue healing and formation of the mineralized barrier cannot be expected unless the infection is controlled. [33]. In our case reports, the mineralized bridge formation underneath MTA was observed radiographically after 12 months in both cases 2 and 3; however, no clear bridge formation was observed in case 1. Although the mineralized bridge formation beneath the capping material is seen as a favorable result in the context of VPT, its radiographic absence is not necessarily regarded as a negative.

Assessing mineralized bridge formation from overlapping two-dimensional radiographs is challenging [34] because of the possibility of overlaying with the restoration or other structures. Furthermore, a dentine bridge that is less than 0.5 mm thick would not be visible on a radiograph [35].

In this case report, all teeth were evaluated every six months for four years, taking into consideration the restoration quality and potential need for additional restorative interventions. The European Society of Endodontology recommends in its statement that VPT should be assessed for 4 years [11]. In addition, the type and quality of the restoration may influence the clinical outcome of pulpotomy over the long term and act as predictors of late failure. [36, 37]. All teeth in the present case reports were restored with composite resin restorations, which maintained their quality throughout follow-up periods. However, two out of three cases needed an additional restorative procedure after two and three years as a result of new carious lesions that were not associated with any signs or symptoms indicating damage to the pulp.

There are not a large number of studies that evaluated the efficacy of partial pulpotomy in the management of irreversible pulpitis during long follow-up. According to Uesrichai et al., the success rate of partial pulpotomy in the management of irreversible pulpitis was 90% in patients between 6 and 18 years old (immature and mature molars) during 36 months [21]. On the other hand, Careddu and Duncan's study have concluded a success rate of more than 90% when using the partial pulpotomy technique on mature permanent teeth (reversible and irreversible pulpitis) during 12 months [19]. Taha and Khazali also found that the MTA partial pulpotomy success rate in the management of irreversible pulpitis reached 83% in mature permanent teeth over a 2-year follow-up [20].

The irrigant with 2.5% NaOCl should be done carefully because of its high cytoxicity, which could lead to damage to pulp tissues in high concentrations, and this is a major limitation of this study. Moreover, MTA mixing could alter the physicochemical properties; it should be mixed according to the manufacturer's instructions.

In conclusion, partial pulpotomy using MTA may be considered a successful treatment in the management of mature permanent teeth with symptoms indicating irreversible pulpitis. However, more clinical studies with larger sample sizes are still required.

## Data Availability

The data used to support the findings of this study are included within the article.
